# Impact of Artificial Intelligence on Pancreaticobiliary Endoscopy

**DOI:** 10.3390/cancers17030379

**Published:** 2025-01-24

**Authors:** Aryan Jain, Mayur Pabba, Aditya Jain, Sahib Singh, Hassam Ali, Rakesh Vinayek, Ganesh Aswath, Neil Sharma, Sumant Inamdar, Antonio Facciorusso

**Affiliations:** 1 Department of Gastroenterology, Albany Medical College, Albany, NY 12208, USA; jaina5@amc.edu (A.J.); pabbam@amc.edu (M.P.); adityaj1000@gmail.com (A.J.); 2 Department of Internal Medicine, Sinai Hospital of Baltimore, Baltimore, MD 21215, USA; 3 Department of Gastroenterology, ECU Health Medical Center/Brody School of Medicine, Greenville, NC 27834, USA; hassamali155@gmail.com; 4 Department of Gastroenterology, Sinai Hospital of Baltimore, Baltimore, MD 21215, USA; rvinayek@hotmail.com; 5 Department of Gastroenterology, State University of New York Upstate Medical University, Syracuse, NY 13210, USA; aswathg@upstate.edu; 6 Department of Gastroenterology, Indiana University School of Medicine, Indianapolis, IN 46202, USA; nrsharma219@yahoo.com; 7 Department of Gastroenterology, University of Arkansas for Medical Sciences, Little Rock, AR 72205, USA; sumant.c.inamdar@gmail.com; 8 Gastroenterology Unit, Department of Experimental Medicine, University of Salento, 73100 Lecce, Italy; antonio.facciorusso@virgilio.it; 9 Clinical Effectiveness Research Group, Faculty of Medicine, Institute of Health and Society, University of Oslo, 0373 Oslo, Norway

**Keywords:** artificial intelligence, endoscopic ultrasound, cholangioscopy, pancreaticobiliary disorders, diagnostic accuracy, pancreatic lesions, biliary strictures, cholangiocarcinoma, ethical considerations, data privacy

## Abstract

Pancreaticobiliary diseases can lead to significant morbidity and their diagnoses rely on imaging and endoscopy which are dependent on operator expertise. Artificial intelligence (AI) has seen a rapid uptake in the field of luminal endoscopy, such as polyp detection during colonoscopy. However, its use for pancreaticobiliary endoscopic modalities such as endoscopic ultrasound (EUS) and cholangioscopy remains scarce, with only few studies available. In this review, we delve into the current evidence, benefits, limitations, and future scope of AI technologies in pancreaticobiliary endoscopy.

## Simple Summary

Diseases affecting the pancreas and bile ducts can cause serious health implications and are often challenging to diagnose because they rely on high-quality imaging and specialized procedures performed by skilled doctors. Artificial intelligence (AI) is already being used in some areas of endoscopy, but its role in diagnosing pancreaticobiliary diseases is still in its early stages. In this review, we explore how AI can be applied to advanced techniques like endoscopic ultrasound and cholangioscopy, highlighting its potential advantages, current challenges, and the opportunities it offers for the future. Our goal is to provide insights into how AI might improve accuracy and efficiency to these procedures, ultimately benefiting patients and shaping the future of pancreaticobiliary care.

## 1. Introduction

Pancreaticobiliary disorders can cause severe morbidity and mortality if not recognized and treated early. Diagnostic assessment relies heavily on imaging modalities and endoscopic procedures, such as cholangioscopy and endoscopic ultrasonography (EUS), which require high skill and experience to interpret accurately [1]. Variations in operator proficiency can result in inconsistent diagnostic outcomes, highlighting the need for innovations that enhance accuracy and standardize care [2].

Artificial intelligence (AI) has revolutionized gastrointestinal endoscopy, particularly in luminal applications like colonoscopy, by improving the detection and characterization of colonic polyps and colorectal cancer [3]. However, its use in pancreaticobiliary endoscopy remains limited, with fewer studies exploring its potential [4].

According to recent studies, AI may enhance diagnostic outcomes in pancreaticobiliary endoscopy. For example, AI-assisted image processing in EUS shows promise for detecting cystic lesions and pancreatic masses and differentiating between benign and malignant conditions [5,6]. AI also holds potential for improving the detection of bile duct strictures and early-stage cholangiocarcinoma when integrated with cholangioscopy, though these results are still preliminary [7]. The complex anatomy and technical difficulty of pancreaticobiliary procedures, which need real-time interpretation of high-resolution images, are obstacles to adopting AI in these procedures. Algorithms that concentrate on EUS-guided fine-needle aspiration (EUS-FNA) and fine-needle biopsy (EUS-FNB) to aid cytological and histological diagnosis are addressing these issues with encouraging outcomes [8,9].

**Figure 1:**
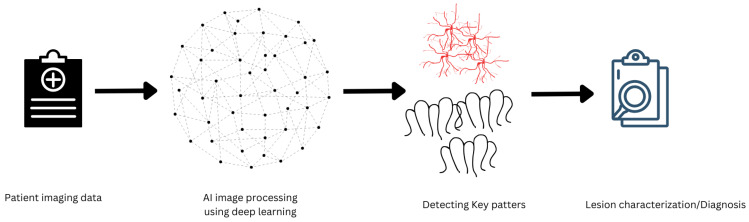
Workflow of AI-Assisted Pancreaticobiliary Endoscopy. [Icons used in these figures were generated in Canva, credited to Bolakaretstudio, Alright2002, Iconsy, and Alyssa Babassa].

**Figure 2:**
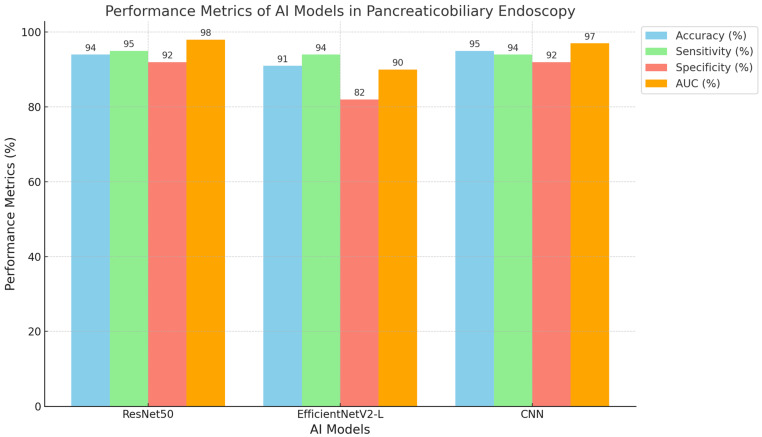
Performance Metrics of AI Models in Pancreaticobiliary Endoscopy.

**Figure 3:**
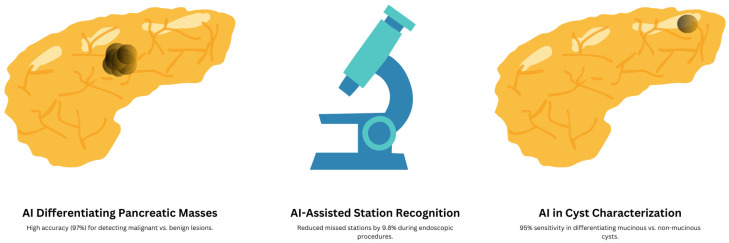
Applications of AI in Pancreaticobiliary Endoscopy. [Icons used in these figures were generated in Canva, credited to Alyssa Babassa].

This study aims to assess the use of AI in pancreaticobiliary endoscopy, with an emphasis on its potential to increase diagnostic and therapeutic accuracy in the future as well as its uses and limitations (Figure 1, Figure 2 and Figure 3).

## 2. EUS

### 2.1. Overview of Disorders Detected by EUS in Pancreaticobiliary Disease

Endoscopic Ultrasound (EUS) is a notable diagnostic modality that aids in evaluating several pancreaticobiliary diseases, offering high-resolution images of the pancreas, bile ducts, and surrounding structures [10]. It is responsible for determining the location, origin, and nature of lesions, crucial factors that help guide the appropriate clinical management plan [11]. EUS-guided fine needle biopsy may be further used for pathological diagnosis and therapeutic purposes [11].

Pancreatic disorders detected by EUS include both inflammatory conditions, such as acute and chronic pancreatitis, and pancreatic masses. Pancreatic masses may be cystic (i.e., intraductal papillary mucinous neoplasm (IPMN), mucinous cystic neoplasm) or solid (i.e., pancreatic ductal adenocarcinoma (PDAC), pancreatic neuroendocrine tumors (PNETs), pancreatic adenosquamous carcinomas) [12,13]. Biliary disorders are similarly classified into inflammatory conditions (i.e., primary sclerosing cholangitis), non-neoplastic lesions (i.e., cholesterol polyps), and neoplastic lesions, with cholangiocarcinoma being the most common malignancy [14,15].

### 2.2. Diagnostic Challenges in EUS

Despite its advantages, EUS faces several diagnostic challenges, particularly in pancreaticobiliary diseases. While it is a primary modality for detecting these conditions, its diagnostic accuracy is not perfect, with estimates ranging from 80% to 95% [16]. This is especially problematic for pancreatic cancer, which it is projected to become the second leading cause of cancer-related mortality in the United States by 2030 [6]. The high mortality rate of pancreatic cancer is partially due to the lack of reliable screening and effective diagnostic options for early detection, so achieving improvements here is critical. Currently, EUS is superior to CT for detecting pancreatic lesions ≤1 cm but still struggles to reliably differentiate benign from malignant lesions [17]. In particular, for pancreatic cystic lesions (PCLs)—where accurate risk stratification is critical—EUS, even with fine-needle aspiration (FNA), achieves only 65–75% accuracy in identifying mucinous PCLs [6]. This diagnostic uncertainty can have profound implications for patient management, given the high risk of malignancy associated with certain PCLs. Furthermore, a prospective study of 115 patients with focal pancreatic lesions reported EUS achieving a high sensitivity of 95% for detecting malignancy but a specificity of only 53% due to overlapping features, such as in the case of chronic pancreatitis [18].

These findings illustrate that EUS struggles to reliably differentiate benign from malignant pancreatic lesions, highlighting the need for improved diagnostic adjuncts. As a result, contrast-enhanced EUS (CE-EUS) and EUS elastography have become more utilized, with an increased specificity of 80% for diagnosing pancreatic cancer [19]. Even with these additions, misdiagnosis is still common because EUS is a highly operator-dependent procedure [20]. The interpretation of images along with the mastery of obtaining high-quality images varies from clinician to clinician based on experience and technical skills. A multicenter study indicated that EUS has a diagnostic accuracy ranging from 65% to 95%, depending on the operator’s experience [20]. Other studies show that inadequate detection and misdiagnosis of masses still arise even if the operator is an expert [21]. These discrepancies are likely attributable to the subjective nature of EUS interpretation, which varies even more in resource-poor settings with less skilled personnel and higher operating costs [22]. This variability can lead to continual inconsistent diagnoses and missed opportunities for early intervention, such as in the case of small pancreatic tumors or subtle biliary obstruction [20]. As a result, the challenges posed by operator dependency and interobserver variability in EUS diagnosis emphasize the need for further standardization of EUS procedures and the use of advanced technology, such as AI, to improve diagnostic accuracy.

### 2.3. AI Models and Techniques in EUS

Advanced technologies, like AI, allow for the possibility of mitigating human error by providing consistent interpretations and enhanced accuracy of EUS images. Broadly, AI refers to the application of computational models to perform tasks, such as classification and regression [23]. In the medical field, AI is used to support ongoing tasks like image diagnosis (CADX) or lesion detection (CADe) using so-called computer-aided detection systems [14]. Several AI technologies have already been integrated into EUS procedures to improve diagnostic performance. These include traditional machine learning (ML) models and more advanced deep learning (DL) algorithms. Machine learning is a subset of AI that depends on algorithms to identify patterns from data and make predictions or classifications based on those patterns [14]. They are used in EUS applications for tasks, such as identifying patterns in pancreatic lesions or distinguishing benign from malignant tumors. They include a range of model architectures, including support vector machines, decision trees, random forests, factorization machines, logistic regression analyses, and neural networks (NNs) [14]. Radiomics is another tool that involves feature extraction from images followed by ML to quantify image characteristics [14]. Deep learning, on the other hand, is an advanced subset of ML. DL uses artificial neural networks (ANNs) with multiple layers to analyze complex data like medical imaging [23]. Unlike ML, DL does not require feature extraction and the selection of thousands of images. Instead, it can directly analyze raw images as input values, making it ideal for EUS applications [23]. One of the most common model architectures of DL is convolutional neural networks (CNNs). CNNs have become the backbone of AI-based image analysis due to their ability to extract complex features from raw image data [24]. As a result, this model has been particularly successful in distinguishing between benign and malignant pancreatic lesions [24]. Of note, ResNet50 and EfficientNet are some of the common CNN architectures used to provide robust frameworks for accurate lesion classification [16,25].

### 2.4. Role of AI in Addressing EUS Diagnostic Challenges

As discussed, the use of EUS is associated with significant variability, underscoring the need for AI to enhance consistency, particularly in classifying lesions, detecting pancreatic parenchyma, and recognizing anatomical stations. AI has emerged as a transformative tool in addressing diagnostic challenges in pancreaticobiliary diseases by improving the accuracy and reliability of EUS. Its applications include the ability to differentiate benign and malignant lesions, enhance the characterization of cystic lesions, assess biliary obstructions, provide real-time diagnostic support, refine risk stratification, and aid in station recognition [15]. By employing these applications, AI can provide an earlier and more precise diagnosis while enhancing prognostic assessments [15]. Ultimately, these advancements aim to mitigate diagnostic errors, streamline workflows, and improve patient outcomes.

#### 2.4.1. Station Recognition and Real-Time Training

During an EUS, accurate station recognition is required to ensure a complete and thorough procedure. It is considered the cornerstone of an effective EUS, as it enables for the precise localization of anatomical landmarks and thorough examination of critical regions [15]. When trying to accomplish comprehensive scanning, two common blind spots commonly arise: the lack of standardization in EUS procedures and the inherent difficulty of interpreting ultrasonographic images [26]. To address these challenges, AI has shown significant potential in enhancing station recognition by standardizing EUS procedures, monitoring blind spots, and simplifying real-time image interpretation [26]. AI algorithms have accomplished this goal by perfecting the ability to recognize and classify anatomical stations. One study conducted by the Renmin Hospital of Wuhan University sought to optimize AI’s ability to monitor blind spots and reduce the number of missed stations [26]. Wu et al. (2023) updated their AI device (EUS-IREAD) to not only accurately identify EUS standard stations and reduce the difficulty of image interpretation but also improve its performance in the quality of EUS procedures [26]. The results demonstrated that the EUS-IREAD-assisted group had a markedly lower rate of missed stations compared to the control group (4.5% [SD 0.8] vs. 14.3% [SD 1.0], with a mean difference of −9.8% [95% CI −12.2 to −7.5]; odds ratio 3.6 [95% CI 2.6 to 4.9]; *p* < 0.0001) [26]. These advancements in station recognition can pave the way for more reliable and reproducible biliopancreatic EUS procedures.

Another application of AI algorithms to enhance the quality of EUS is the use of real-time feedback. AI-powered systems developed the technology to offer real-time feedback during EUS procedures [15]. This is advantageous to clinicians as it allows for immediate diagnostic assistance during the procedure. The use of real-time feedback can act as a great tool for endoscopists still in training. For example, the use of CE-EUS is recommended to achieve early detection of solid pancreatic masses; however, the procedure is notably difficult to learn [27]. As a result, Tang et al. (2023) developed CH-EUS MASTER, a deep learning-based system for real-time capture and segmentation of solid pancreatic masses during CH-EUS [27]. The results displayed success in the segmentation of pancreatic masses (Dice coefficient of 0.763, recall rate of 0.941, and an accuracy of 0.842) [27]. Moreover, the AI system improved training outcomes through the use of CH-EUS MASTER; there was a significant improvement in their segmentation performance (average IoU increased from 0.80 to 0.87; 95% CI, 0.032–0.096; *p* = 0.002) and it reduced the time needed to identify pancreatic lesions in the body, tail, head, and uncinate process (*p* < 0.01) [27]. These findings highlight the role of AI in not only enhancing real-time diagnostic precision but also expediting clinician training, thereby improving procedural efficiency and diagnostic accuracy.

#### 2.4.2. Improving the Differentiation of Pancreatic Masses

One of the main advancements with AI in EUS is its ability to precisely differentiate pancreatic masses. A significant challenge lies in distinguishing pancreatic cancer from benign or non-cancerous conditions, such as chronic pancreatitis and PNET [15]. Although these conditions may appear similar on EUS imaging, their management strategies vastly differ. For instance, PDAC is a highly malignant form of pancreatic cancer with a 5-year life expectancy of 11% [28]. Given its poor prognosis, improving the effectiveness of current screening methods is critical. There have been several studies that have examined the effectiveness of AI algorithms to detect and appropriately diagnose pancreatic solid lesions during an EUS. Of note, a randomized crossover trial in China displayed AI’s ability to aid in diagnosing solid lesions in the pancreas. The study introduced a multimodal AI model that integrated clinical information with EUS imaging, significantly enhancing diagnostic accuracy. Novice endoscopists, in particular, saw a substantial improvement in performance, with their diagnostic accuracy increasing from 69% to 90% when assisted by AI (*p* < 0.001) [29]. Similarly, another study highlighted AI’s potential to classify pancreatic cancer. Ozkan et al. (2016) designed a high-performance CAD system to enhance image processing and pattern recognition for diagnosing pancreatic cancer using endosonography images [30]. Using an artificial neural network (ANN), the study reported improvements in sensitivity, specificity, and accuracy across all age groups, with values of 83.3%, 93.3%, and 87.5%, respectively [30]. Expanding on the potential of AI to enhance diagnostic accuracy, another study explored the use of digital image processing (DIP) techniques to classify pancreatic cancer using EUS images [31]. Zhang et al. (2010) developed a predictive model by extracting texture features from EUS images and correlating them with cytologic findings from FNA via a support vector machine [31]. The study achieved an impressive diagnostic accuracy of 97.98%, with a sensitivity of 94.32% and a specificity of 99.45%, suggesting that DIP could significantly improve the accuracy of pancreatic cancer diagnosis using EUS [31].

##### Chronic and Autoimmune Pancreatitis vs. Pancreatic Cancer

In addition to examining the effectiveness of classifying pancreatic cancer, many researchers have specifically investigated AI’s capacity to distinguish pancreatic cancer from chronic pancreatitis. Although EUS has proven effective in differentiating PDAC from other benign conditions, chronic pancreatitis poses significant diagnostic challenges due to its similar imaging characteristics [32]. Specifically, scars and calcifications resulting from chronic inflammation can make it particularly difficult for less experienced endoscopists to distinguish it from pancreatic cancer [24]. One prospective, multi-center study attempted to compare the diagnostic performance of EUS-FNA, CEH-EUS, and ANN classification in differentiating pancreatic cancer from chronic pancreatitis. Săftoiu et al. (2015) evaluated the performance based on post-processing of CEH-EUS recordings and found that ANN classification produced better results, with a sensitivity of 94.64%, specificity of 94.44%, PPV of 97.24%, and NPV of 89.47%, compared to EUS-FNA (sensitivity 84.82%, specificity 100%, PPV 100%, NPV 76.63%) and CEH-EUS (sensitivity 87.5%, specificity 92.72%, PPV 96.07%, NPV 78.46%) [33]. Another similar multicenter study, this time with EUS-elastography, demonstrated impressive results of ANN classification in distinguishing pancreatic cancer versus chronic pancreatitis, achieving a sensitivity of 87.59% and specificity of 82.94%. This outperformed two experienced endoscopists, who had sensitivities of 84.4% and 75.4%, and specificities of 46.8% and 53.2%, respectively [32]. Building on these advancements, another notable multicenter study by Săftoiu et al. (2011) explored the use of real-time EUS elastography combined with ANN classification to further refine the differentiation of pancreatic adenocarcinoma from chronic pancreatitis. By leveraging hue histogram data and utilizing neural networks (NNs), this study sought to provide a more robust AI-supported decision-making tool. It was found that the ANN achieved a sensitivity of 87.59%, a specificity of 82.94%, and an area under the ROC curve of 0.94, outperforming the performance of hue histogram analysis (ROC area of 0.85) [34]. These findings suggest the potential of ANN in enhancing diagnostic accuracy between chronic pancreatitis and pancreatic cancer. Conversely, a recent study by Kuwahara et al. (2023) utilized a different deep-learning approach, specifically EfficientNetV2-L, to classify EUS images from various pancreatic diseases, including pancreatic cancer, autoimmune pancreatitis, and chronic pancreatitis. Their AI model achieved an impressive diagnostic accuracy of 91%, surpassing both the preoperative diagnostic accuracy (58%) and EUS image findings (52–68%) [16]. This deep learning model approach may offer more robust diagnostic capabilities by providing more conclusive and reliable findings. Similar to chronic pancreatitis, autoimmune pancreatitis presents as a challenge when diagnosing pancreatic cancer. Sonographic and cross-sectional images of autoimmune pancreatitis closely resemble those of PDAC [24]. Moreover, current techniques for tissue sampling of autoimmune pancreatitis are suboptimal. To combat this issue, Marya et al. (2020) utilized a deep learning model approach to develop a CNN (ResNet50v2) that appropriately differentiates autoimmune pancreatitis from PDAC, chronic pancreatitis, and a normal pancreas. They reported a sensitivity of 90% and specificity of 85% for distinguishing autoimmune pancreatitis from other conditions, demonstrating the potential of deep learning models in improving diagnostic accuracy [24].

##### Pancreatic Neuroendocrine Tumors (PNET) vs. PDAC

Along with autoimmune pancreatitis and chronic pancreatitis, PNET is another disorder that closely resembles the highly malignant PDAC on EUS imaging. This is primarily due to the hypovascular patterns shown for PNET that are also displayed for PDAC [35]. PNET not only closely resembles PDAC, but it also can mimic other pancreatic conditions, such as autoimmune pancreatitis, pancreatic cystic neoplasm (PCN), and even a normal pancreas [36]. Distinguishing these disorders is essential as they require different management plans [15]. To improve this specific diagnostic ability, Ni et al. (2024) conducted a recent study developing a CNN, namely iEUS, to precisely differentiate pancreatic neuroendocrine neoplasm (pNEN) from other pancreatic conditions. Their findings reported that iEUS demonstrated accuracy rates of 84.2% and 88.2% for diagnosing pNEN using two different models: CNN1, which classified lesions into two categories (pNEN vs. non-pNEN), and CNN2, which differentiated four categories (pNEN, PDAC, AIP, and PCN) [36]. These models significantly outperformed novices and revealed that iEUS notably improved sensitivity at all levels of experience in diagnosing pNEN, highlighting AI’s potential as a valuable tool in EUS.

#### 2.4.3. Enhancing Cystic Lesion Characterization

##### Differentiating Between Mucinous and Non-Mucinous Pancreatic Cysts

Pancreatic cystic lesions are increasingly recognized as a significant subset of pancreatic disorders and are notable for their diverse clinical implications. These lesions can be broadly classified into mucinous and non-mucinous cystic lesions, each with distinct malignant potential. Mucinous cystic lesions, such as intraductal papillary mucinous neoplasm (IPMN) and mucinous cystic neoplasm (MCN), carry a higher risk of malignancy and often require surgical intervention or close surveillance [37]. In contrast, non-mucinous cystic lesions, including serous cystic neoplasm (SCN) and pancreatic pseudocyst (PPC), are generally benign and pose minimal malignant risk [38]. Given the substantial differences in prognosis and management strategies for these lesions, optimizing diagnostic modalities, particularly endoscopic ultrasound (EUS), is critical to accurately differentiate mucinous from non-mucinous cystic lesions. Unfortunately, current imaging modalities, like EUS and CT, have struggled to consistently differentiate mucinous from non-mucinous cystic lesions [39]. One recent study found that the accuracy in differentiating mucinous from non-mucinous cystic lesions via the use of EUS significantly varied from 48% to 94%, with a sensitivity ranging from 36% to 91% and specificity varying from 45% to 81% [40]. Recent advancements in artificial intelligence (AI), however, have shown promise in addressing these diagnostic gaps. A notable study by Kurita et al. (2019) investigated the diagnostic ability of AI, carcinoembryonic antigen (CEA), and cytology in differentiating malignant from benign pancreatic cystic lesions. By integrating cyst fluid markers (CEA, carbohydrate antigens (CA 19-9, CA 125), amylase levels) and clinical features into a deep learning algorithm, the AI was able to achieve a remarkable diagnostic accuracy of 92.9%, with a sensitivity and specificity of 95.7% and 91.9%, respectively. These results significantly outperformed traditional methods, especially when comparing the sensitivities (CEA- 60.9%, *p* = 0.021, cytology- 47.8%, *p* = 0.001). Additionally, the AI algorithm demonstrated an area under the receiver operating characteristic curve (AUC) of 0.966 [41]. Another study developed a CNN algorithm to automatically diagnose mucinous cystic lesions. Boas et al. (2022) discovered that their AI model, trained on 5505 endoscopic ultrasound (EUS) images, achieved an impressive accuracy of 98.5%. The CNN demonstrated a sensitivity of 98.3%, a specificity of 98.9%, and an AUC of 1 [42]. These findings further underscore AI’s potential to improve the diagnostic accuracy of pancreatic cystic lesions.

##### Enhancing Diagnosis of Malignant IPMN

Among the neoplastic pancreatic cystic lesions, IPMN is the most frequent and difficult to diagnose [43]. It is a type of mucin-producing cystic lesion of the pancreas, with a known potential for malignant transformation [25]. IPMN is a difficult disorder to diagnose because its lesions can range from benign to malignant forms. Malignant IPMN lesions often require surgical resection, whereas benign forms may be managed conservatively or with surveillance [25]. Current imaging techniques, including EUS, have shown limited sensitivity and specificity for the diagnosis of malignant IPMN [44]. To enhance predictive accuracy and guide clinical decisions, several studies have demonstrated AI’s successful role in diagnosing malignant IPMN lesions during an EUS. Kuwahara et al. (2019) developed a deep learning architecture, ResNet50, to differentiate benign and malignant lesions of IPMN. Using EUS images from 50 IPMN cases, they achieved a mean AI value of 0.808 for malignant lesions versus 0.104 for benign (*p* < 0.001). Additionally, the AI model achieved an AUC of 0.98, sensitivity of 95.7%, specificity of 92.6%, and accuracy of 94.0%, surpassing the accuracy of human diagnosis (56.0%) and mural nodules (68.0%). AI was found to be the only independent factor to predict malignancy (odds ratio: 295.16, *p* < 0.001) [25]. On the contrary, Machicado et al. (2021) explored the potential of AI to enhance the diagnostic ability of EUS-guided needle-based confocal laser endomicroscopy (EUS-nCLE) in detecting malignant IPMN lesions. While EUS-nCLE is known to differentiate high-grade dysplasia/adenocarcinoma (HGD-Ca) in IPMNs, it traditionally requires manual interpretation. As a result, they developed two CNN-based CAD models: The first model, a guided segmentation-based model (SBM), was trained to detect and measure papillary epithelial thickness and darkness, which are key features for identifying high-grade dysplasia or adenocarcinoma. The second model, a holistic-based model (HBM), was designed to automatically extract and analyze various nCLE features across the entire lesion for effective risk stratification, thereby improving the accuracy of diagnosing malignant lesions without the need for manual interpretation. Their study reported that both the SBM and HBM CNN-CAD algorithms demonstrated higher sensitivity (83.3%) and accuracy (82.9% and 85.7%, respectively) for detecting HGD-Ca in IPMNs compared to the AGA (55.6%, 68.6%) and Fukuoka guidelines (55.6%, 74.3%) [45]. These findings highlight the potential of AI in EUS to accurately diagnose the complex benign/malignant nature of IPMN.

#### 2.4.4. Diagnostic Performance of AI in Differentiating Gallbladder Polyps Using EUS

Polypoid lesions of the gallbladder (GB) are elevated growths of the mucosal wall that protrude into the GB lumen, encompassing a range of pathologies [46]. They include non-neoplastic lesions such as cholesterol polyps, inflammatory polyps, and adenomyomatosis, as well as neoplastic lesions like adenoma and adenocarcinoma. The differential diagnosis of GB polyps remains challenging, as these lesions cannot be biopsied. EUS is currently the most accurately used diagnostic modality for this illness; however, it is still limited to subjective interpretation. Jang et al. (2021) sought to improve the diagnostic performance of EUS in diagnosing polypoid lesions of the GB through the utilization of a deep learning architecture (ResNet50). The diagnostic performance of EUS with the ResNet50 architecture was found to be promising, with a sensitivity of 57.9%, specificity of 96.5%, and accuracy of 89.8% in the AI development cohort, and comparable accuracy between mid-level (66.7%) and expert (77.5%) EUS endoscopists in the external validation cohort [46]. These results showcase AI’s potential in enhancing the diagnosis of neoplastic GB lesions during an EUS, particularly when compared to other expert-level endoscopists. Table 1 provides an overview of the key studies that tested AI models in EUS to aid in the diagnosis of pancreaticobiliary disorders; it summarizes their objectives, diagnostic outcomes, and AI model performance.

### 2.5. Future Directions of AI in EUS

The observed studies provide only a glimpse of how AI can positively influence the diagnosis of pancreaticobiliary disorders through the use of EUS. There remain significant opportunities and advancements to be explored to optimize diagnosis accuracy. One key advancement is the standardization of diagnosis with AI-assisted EUS. Although AI-assisted EUS has demonstrated improved diagnostic accuracy, achieving 100% diagnostic accuracy remains elusive. By enabling specialists to utilize AI systems that reduce interobserver variability and continuously refine their performance, standardization could ultimately enhance diagnostic consistency and improve patient outcomes [47]. Furthermore, AI advancements could enhance real-time EUS by enabling algorithms to accurately identify optimal biopsy sites during procedures, thereby minimizing the risk of complications and improving procedural outcomes [15]. Additional research with larger data sets is still required for AI to improve generalizability and optimize real-time feedback during an EUS [48]. Risk stratification is another promising application of AI in pancreaticobiliary endoscopy that can enhance early detection and guide treatment strategies. Even though EUS screening has displayed a favorable 73% 5-year survival in several cancers, only a minority of cancers are detected in this manner [28]. As a result, it is critical to integrate AI in early detection outcomes through the use of biomarkers and risk models. For example, the identification of specific biomarkers in pancreatic ductal adenocarcinoma (PDAC) serves as a critical indicator for disease progression and prognosis [28]. AI algorithms could be designed to analyze these biomarkers in conjunction with patient risk factors, enabling the ability to stratify patients into appropriate risk groups [15]. Ultimately, AI’s capacity to analyze large EUS datasets, identify patterns, and evaluate treatment responses has the potential to refine diagnoses and facilitate the development of innovative therapies for pancreaticobiliary disorders.

### 2.6. Limitations of AI in EUS

The integration of AI into EUS for pancreaticobiliary diseases promises to revolutionize diagnostic practices by improving accuracy, reducing operator dependency, and facilitating early detection of malignant lesions. However, some limitations remain. One such limitation is the need for large, diverse datasets to ensure that AI models generalize well across different patient populations [22]. This is further displayed when examining rare pancreatic conditions. These studies are generally retrospective and utilize a limited number of images from single-center studies, limiting the generalizability of the findings. Due to the scarcity of comprehensive datasets for rare pancreatic conditions, AI models may struggle with overfitting, reducing their effectiveness in real-world clinical settings [49]. Additionally, there is a lack of external validation in many studies, especially with AI models used for EUS in the pancreas [22,28]. In one study examining image-based diagnostic AI study designs, only 6% of image-based diagnostic AI studies included external validation, which hinders the clinical applicability and robustness of AI systems [50]. To address these challenges, AI development must prioritize diverse, multi-center datasets to ensure the accuracy and applicability of models across varied patient populations. It is also essential to employ collaborative efforts and data sharing among hospitals and academic institutions to overcome the limitations of small, single-center datasets [22]. By conducting robust multicenter trials, researchers can increase the sample size, thereby improving the statistical power and clinical relevance of study results. Additionally, the application of AI in pancreaticobiliary endoscopy comes with the potential for selection bias and misclassification. This has led to several deep learning models producing suboptimal performance of CNNs [22]. Moreover, bias in AI model building has led to the lack of inclusivity of underrepresented minorities, disadvantaged socioeconomic groups, and rare conditions [28].

Lastly, there are several ethical, legal, and regulatory challenges in applying AI systems to EUS. One major concern is data privacy, as the use of AI in healthcare involves collecting sensitive patient data, raising issues of patient consent and confidentiality [22]. AI models require substantial amounts of patient data, including images and videos from procedures, for training and validation purposes [51]. Collecting and storing this sensitive data raises concerns about patient privacy and the risk of unauthorized access or disclosure. Additionally, real-time training of AI models poses ethical and safety concerns, as the system may adapt based on unvalidated data [51,52]. Unlike physicians, AI cannot explain its reasoning, which can lead to situations where the physician is pressured to agree with the AI’s output or, conversely, dismiss it without adequate justification. This can impair clinical judgment and affect patient care, which is why developers need to focus on making AI models more transparent and easier to interpret [53]. The reliance on AI also raises important legal questions: when AI makes an incorrect diagnosis or fails to detect a lesion, who should be held responsible? Is the blame on the physician, the AI developer, or the AI itself? Current regulations by the FDA suggest that the physician carries ultimate responsibility, as AI-based diagnostic tools are classified as “Software as a Medical Device” (SaMD); this means they are meant to only assist in detection and diagnosis [51]. However, as AI-based diagnosis continues to become more powerful and accurate, the regulations will need to evolve and become clearer. To address these ethical concerns, clear guidelines and regulations must be established to govern AI use in healthcare, ensuring data privacy, transparent processes, and a balanced integration of AI and physician expertise.

## 3. Cholangioscopy

### 3.1. Introduction to Cholangioscopy

A biliary stricture is a narrowing of the bile duct that prevents bile from flowing from the liver into the small intestine. A malignant biliary stricture (MBS) is caused by a malignant tumor that obstructs the flow of bile through the duct; they are the most common type of biliary stricture (70–80% of all biliary strictures) and are caused by a primary neoplasm of the bile duct (cholangiocarcinoma) or a secondary neoplasm with biliary tract extension, such as hepatocellular, pancreatic, or gallbladder carcinoma [54,55]. Benign strictures are often caused by other factors, such as biliary lithiasis and sclerosing cholangitis, and their treatment thus depends on the specific cause [55]. MBSs are often associated with a poor prognosis, as they are typically diagnosed at a late stage when they can no longer be cured with resective surgery [55]. Due to the vastly different treatment and prognosis of benign and malignant biliary strictures, accurate diagnosis is critical. Therefore, when a biliary stricture is suspected, the primary objective is to rule out an MBS [55,56].

There are several diagnostic imaging tools used to diagnose and differentiate between benign and malignant biliary strictures. Cholangioscopy is a procedure that allows physicians to visualize the biliary tract using a thin and flexible tube that is attached to a camera and light. Cholangioscopy can be performed through various approaches, including peroral, percutaneous, and surgical access, depending on the clinical indication and the location. This procedure offers a more detailed and direct view of the bile ducts compared to older imaging methods such as endoscopic retrograde cholangiopancreatography (ERCP), which only provides indirect images [54,57]. ERCP, combined with brush cytology or biopsy, is a standard procedure used in biliary stricture diagnosis, as it has a high specificity (up to 99%) [54]. However, its sensitivity is less than 50%, making it less effective in differentiating malignant and benign biliary strictures [3,54]. Digital single-operator cholangioscopy (DSOC) is a newer form of cholangioscopy that produces higher-resolution images of the biliary tract; it has a higher sensitivity and specificity than ERCP [54]. Thus, the introduction of DSOC has led to the increased utilization of cholangioscopy in endoscopy centers worldwide [54].

### 3.2. Diagnostic Challenges

Despite the improvements in cholangioscopy, there are still considerable challenges in interpreting its images. Differentiating between benign and malignant biliary strictures is still a diagnostic challenge for physicians, as it varies significantly depending on their individual judgment and expertise [3]. Up to 10% of malignant biliary strictures were missed in DSOC, even when accounting for the use of direct visualization or targeted biopsies [55]. Additionally, despite its improvements to the diagnostic accuracy of MBSs, the sensitivity of DSOC is still only approximately 70% [56]. This is where AI can be used to aid in the effectiveness of cholangioscopy, as it does not rely on the subjective interpretation of the physician. By improving diagnostic accuracy and efficiency, AI has the potential to revolutionize the diagnosis and treatment of biliary strictures.

Cholangiocarcinoma (CCA) is a malignant bile duct cancer that arises from epithelial cells of the bile ducts [58]. CCA usually occurs due to primary sclerosing cholangitis, hepatobiliary flukes, Caroli’s syndrome, or congenital hepatic fibrosis [7]. Like MBS, cholangiocarcinoma can be cured by surgical resection of the lesions before they spread. However, also like MBS, cholangiocarcinoma often has a poor prognosis because most patients are diagnosed at an advanced stage; in fact, 5-year survival rates from CCA after surgery are often below 35% [59,60].

### 3.3. AI Models and Techniques in Cholangioscopy

#### 3.3.1. CNN Models

AI has been utilized to aid in the diagnosis of malignant biliary strictures and cholangiocarcinoma, with convolutional neural networks (CNNs) being an early form of machine learning implemented in diagnostic efforts. CNNs are fed large collections of cholangioscopy images, which trains their pattern recognition ability. CNNs have shown promising results, with this pilot study by Saraiva et al. (2022) finding that CNN-cholangioscopy performed very well in the identification of MBSs, with an accuracy of 94.9%, sensitivity of 94.7%, and specificity of 92.1%, and AUC of 0.988 in cross-validation analysis [56]. The study concluded that the deep learning algorithm “accurately detected and differentiated malignant strictures from benign biliary conditions”, suggesting that it could be effectively implemented in DSOC usage to improve diagnostic yield [7]. Another study by Marya et al. (2023) compared the accuracy of a CNN model with traditional ERCP-based brush cytology and tissue biopsy methods in identifying and classifying malignant biliary strictures. Their CNN model was trained using over 2 million cholangioscopy still images from 154 different patients, and this training proved effective, as the CNN model achieved an overall accuracy of 90.6%, significantly higher than brush cytology (62.5%) and forceps biopsy sampling (60.9%) [58]. Robles-Medranda et al. (2023) developed a DSOC-AI CNN model, especially for identifying malignancies from indeterminate biliary strictures that were difficult to diagnose [61]. Their DSOC-AI model demonstrated similar diagnostic accuracy when compared to regular DSOC and DSOC-guided biopsies [61]. Zhang et al. (2023) developed an AI model called MBSDeiT that identified MBSs from images with an AUC between 0.971 and 0.999, which exceeded the accuracy of expert and novice-level endoscopists significantly [54]. Along with the CNN model designed by Saraiva et al. (2023), this MBSDeiT model was one of the most promising implementations of AI in diagnosing malignant biliary strictures and cholangiocarcinomas from cholangioscopy images. Overall, a review of many CNN models has found that many cholangioscopy AI models outperformed traditional ERCP imaging methods and expert endoscopists, all while processing each image frame within 7–15 milliseconds [7,56,58,62,63]. These findings suggest the promising potential of AI in digital single-operator cholangioscopy, as it could improve patient outcomes by leading to more accurate and earlier diagnoses. Table 2 provides an overview of the key studies employing CNNs in cholangioscopy, summarizing their objectives, diagnostic outcomes, and AI model performance.

As with other implementations of AI in medicine, AI can be used as a “second-opinion consultation” in cholangioscopy, particularly in difficult cases where there is uncertainty with a diagnosis [64]. This standalone assessment of biliary strictures would be especially valuable in community hospitals and rural practices where physicians have limited expertise in cholangioscopy, as it can quickly and accurately interpret images. AIWorks-Cholangioscopy is an AI-based cholangioscopy image interpreter based in Ecuador, and its successful implementation suggests the potential for AI in this case in the future [64]. The CNN models could also be used as a source of a second opinion in the identification of malignant biliary strictures as well. While AI does have potential as a standalone diagnostic method due to its accuracy in identifying MBSs and CCAs, it would likely best be used as a tool to complement other cholangioscopy-based diagnostic tools; currently, developers do not endorse their AI models’ ability as a standalone diagnostician [57].

#### 3.3.2. Application of AI in Virtual Indigo Carmine Chromoendoscopy

Another way AI is being implemented into cholangioscopy imaging techniques is through virtual indigo carmine chromoendoscopy (VICI). Standard cholangioscopy uses white light to generate images, whereas chromoendoscopy uses a dye to stain the area of interest. The staining helps visualize the surface morphology of epithelial tissue, as it can highlight abnormalities and outline the margins of lesions, which is important in early gastric cancer diagnosis and the detection of dysplastic lesions in inflammatory bowel disease [65]. Virtual chromoendoscopy is an AI-based technology that creates chromoendoscopy images from standard white-light cholangioscopy, which minimizes unnecessary dye-based procedures and enables this enhanced visualization in patients in whom dye-based staining may not be possible, such as individuals with complex biliary stricture and anatomical variations [66]. In addition to these patients where indigo carmine staining may not be possible at all, the presence of bile or saline in the bile duct lumen during the cholangioscopy procedure can make applying the stain even more challenging [66]. Thus, virtual indigo carmine chromoendoscopy has many real clinical applications. VICI has already been used in clinical practice, and it achieved a similar rate of effectiveness when compared to traditional chromoendoscopy with physical indigo carmine spraying [66]. In clinical cases where indigo carmine spraying within the bile duct was difficult, VICI was the preferred diagnostic tool [66].

**Table 2:** Convolutional Neural Networks in Cholangioscopy.

Study	Objective	Diagnostic Metrics	Conclusions
Zhang et al. (2023) [54]	Real-time prediction of MBSs during DSOC	92.3% accuracy,95.6% sensitivity, 89.0% specificity, 0.976 AUC	CNN had a superior performance to expert and novice endoscopists
Saraiva et al. (2023) [55]	Identify MBSs in DSOC and detection of morphologic features (tumor vessels, papillary projections, etc.)	82.9% accuracy, 83.5% sensitivity, 82.4% specificity, 0.92 AUC	Successful in differentiating malignant and benign BSs
Saraiva et al. (2022) [56]	Automatic detection of MBSs in DSOC	94.9% accuracy, 94.7% sensitivity, 92.1% specificity, 0.988 AUC	Duccessful in differentiating malignant and benign BSs
Marya et al. (2023) [58]	Compare CNN detection of MBSs to brush cytology and forceps biopsy sampling	90.6% accuracy, 93.3% sensitivity, 88.2% specificity	CNN was more accurate than ERCP-based methods in BS diagnosis
Robles-Medranda et al. (2024) [61]	Compare differentiation of malignant and benign BSs between DSOC-AI, DSOC-pCLE, DSOC alone	97.7% sensitivity, 75% specificity, 0.790 AUC	DSOC-AI model had a similar performance to DSOC alone and DSOC-pCLE
Ribiero et al. (2021) [62]	Automatic detection of papillary projections in DSOC	98.2% accuracy, 99.7% sensitivity, 97.1% specificity, 1.00 AUC	CNN was able to detect papillary projections with high accuracy
Pereira et al. (2022) [67]	Automatic detection of tumor vessels in DSOC	99.3% accuracy, 99.3% sensitivity, 99.4% specificity, 1.00 AUC	CNN was able to detect tumor vessels with high accuracy

#### 3.3.3. Real-Time Detection of Pancreaticobiliary Disease Using AI

The previous two applications of AI in cholangioscopy focused on the implementation of AI on pre-existing cholangioscopy images. However, one of the most promising applications of AI in cholangioscopy is its real-time detection of patterns of malignant biliary strictures. AI algorithms, like CNN models, can be trained to recognize specific visual cues and patterns associated with MBSs, such as tumor vessels, papillary projections, and abnormal mucosal patterns [54,67,68]. AI algorithms were extremely accurate in distinguishing these malignancy patterns, as they were able to identify tumor vessels with a sensitivity of 99.3%, specificity of 99.4%, positive predictive value of 99.6%, negative predictive value of 98.7%, and AUC of 1.00 [67]. The MBSDeiT model developed by Zhang et al. (2023) accurately identified 92.3% of MBSs in prospective testing videos, which exceeded the performance of both expert and novice-level endoscopists [54]. Their model was effective in predicting MBSs based on the presence of nodular mass, friability, raised intraductal lesion, and abnormal vessels [54]. The real-time detection of these malignancy trends can help endoscopy operators identify and focus on the suspected areas of stricture, reducing the need for repeat procedures and leading to targeted biopsies that can be performed in an area with a high likelihood of malignancy [54,58].

#### 3.3.4. Applications in Medical Education

Another application of AI in cholangioscopy is medical education, as it can be used to train less experienced endoscopy operators in the patterns of MBSs and CCAs [3,64]. AI can create simulated cholangioscopy cases that allow students to practice identifying biliary strictures and make real-time clinical decisions in a simulated environment that does not harm patients. Based on the simulation, the AI training system could provide performance feedback to the students, allowing them to target specific areas of improvement and improve in recognizing the subtle indicators of MBSs, CCAs, and other pancreaticobiliary diseases.

Within medical education, AI can be used to standardize diagnostic criteria of malignant biliary strictures and cholangiocarcinomas in imaging. As stated, the interpretation of visual findings from cholangioscopy images can be subjective, leading to variations in diagnoses across physicians. By using AI to analyze large collections of images, training programs can establish more consistent and objective diagnostic criteria based on the prevalent patterns. This standardization would help reduce interobserver variability, ensuring that all providers are equipped with the same knowledge to make an accurate diagnosis.

All of these potential implementations suggest that cholangioscopy-AI models can have a positive impact in improving the diagnosis, treatment, and management of biliary strictures, cholangiocarcinoma, and other pancreaticobiliary diseases. AI could improve the overall efficiency of cholangioscopy by preventing the need for repeat procedures and making more accurate and earlier diagnoses; this could improve the overall cost-effectiveness of cholangioscopy and lead to better patient outcomes. However, while these results from early studies are very promising, there are still several obstacles and challenges in the widespread implementation of AI in cholangioscopy.

### 3.4. Limitations and Future Directions of AI in Cholangioscopy

There are many limitations to the utilization of AI in cholangioscopy; some are general limitations of AI in healthcare, while a couple are directly related to cholangioscopy due to the AI models that have been developed for cholangioscopy. A common limitation in some pilot studies that develop AI models for cholangioscopy is small sample sizes, which reduces the generalizability of the findings [54,55,62]. Although they may have lots of images, they are often derived from a small group of patients [58]. Moreover, the biggest limitation of these studies is that they lack diversity within the datasets used to train the AI models, as the images tend to come from a single source [3,54,55,56,57,67,68]. This is an issue because it can introduce bias and reduce the external validity of the AI models. Overall, if there is a small sample size and a lack of diversity in images, the training dataset could end up being unrepresentative of the target population, leading to a hindered ability for the AI model to accurately diagnose pancreaticobiliary diseases. However, the small and homogenous samples will not be a limitation forever; the advantage of AI is that it can be trained based on millions of images extremely quickly, whereas a human endoscopist would need years of training to see that many different cases. As the usage of AI in cholangioscopy grows, the sample size and diversity of images will increase, leading to data that are more representative of the target population. Additionally, to test the external validity of the models, future studies can test the AI with datasets different from the ones used to train the model. This would provide a more realistic assessment of the AI model’s ability to diagnose pancreaticobiliary diseases in a clinical setting. All of this will lead to AI models that can more accurately diagnose and treat patients through cholangioscopy.

Another limitation of AI-based cholangioscopy is the application of AI models in clinical practice due to the multiple etiologies of malignant biliary strictures [58]. Although most MBSs are secondary to cholangiocarcinoma, they could also be secondary to gallbladder cancer, lymphoma, neuroendocrine tumors, and more; however, all of these have very different treatment plans [58]. These MBSs can look very similar in images, and since treatment options are not the same, further distinction beyond benign and malignant is necessary. Classification of malignant biliary strictures would be an important future direction for research, as this could increase the efficiency of diagnostic and treatment efforts.

To tackle some of these limitations and continually improve the implementation of AI in cholangioscopy, future research should explore various directions. Improving clinical applicability and accuracy are central focuses of future research. To make findings from AI models more reliable, future studies should try to incorporate clinical information like patient history, blood tests, and other imaging tests into the AI algorithm to improve their diagnostic accuracy [69]. The development of “explainable” AI is also a focus of future research, as it allows physicians to understand the determinants of the AI’s diagnosis [3,70]. This would aid clinical decision-making because physicians can better utilize the AI’s recommendations when making a diagnosis.

Another aspect of future research could be integrating AI-based models with robotic systems to automate aspects of cholangioscopy procedures. Robotics-assisted endoscopy is an extremely precise procedure, and when combined with AI algorithms, endoscopists could automate navigation through the biliary tree and quickly identify areas with a high likelihood of malignancy. This technology would ultimately reduce procedure times and interobserver variability. To achieve the potential of AI-based DSOC, future research must continue to focus on improving the applicability of AI algorithms in real-time procedures.

Overall, AI has the potential to significantly improve the diagnosis and management of biliary strictures. With continued advancements in AI algorithms, data acquisition, and integration with other technologies, AI-based DSOC has the potential to become an essential tool for endoscopists, leading to more accurate diagnoses, more targeted interventions, and improved patient outcomes.

## 4. Conclusions

The application of AI has the potential to change clinical practice in pancreaticobiliary endoscopy, as seen in other fields of medicine [71,72,73,74]. This paper reviewed many pilot studies that tested the implementation of artificial intelligence models in pancreaticobiliary endoscopy, and they showed the potential of AI in helping physicians find lesions during procedures and accurately differentiating between benign conditions and malignancies. By improving diagnostic precision and procedural efficiency, artificial intelligence is poised to transform the field of pancreaticobiliary endoscopy. Although still in the early stages of development, the rapid pace of innovation and ongoing research suggest that AI will play an increasingly important role in the future of pancreaticobiliary care.

## Figures and Tables

**Table 1:** AI Models in EUS Used for Pancreaticobiliary Disorders.

Study	Objective	Diagnostic Metrics	Conclusions
Kuwahara et al. (2023) [16]	Use EfficientNetV2-L to classify EUS images from pancreatic diseases	91% accuracy, 94% sensitivity, 82% specificity, 0.90 AUC	AI model can distinguish pancreatic carcinomas from noncarcinomatous pancreatic lesions
Marya et al. (2020) [24]	Create EUS-based CNN model to differentiate AIP from PDAC, chronic pancreatitis, and normal pancreas	90% sensitivity, 85% specificity, 0.790 AUC	Accurately distinguished AIP from PDAC and benign pancreatic conditions
Ozkan et al. (2016) [30]	Diagnose pancreatic cancer using endosonography through CAD system	87.5% accuracy, 83.3% sensitivity, 93.3% specificity	Diagnoses using classification by patient age were superior to diagnoses without classification
Zhang et al. (2010) [31]	Classify pancreatic cancer using EUS images and DIP techniques	97.98% accuracy, 94.32% sensitivity, 99.45% specificity	Successful in differentiating pancreatic cancer and normal tissue
Săftoiu et al. (2015) [34]	Use real-time EUS elastography with ANN analysis to diagnose pancreatic carcinoma and chronic pancreatitis	87.59% sensitivity, 82.94% specificity, 0.94 AUC	Successful in differentiating pancreatic carcinoma and chronic pancreatitis
Kurita et al. (2019) [41]	Differentiate malignant from benign pancreatic cystic lesions using CEA, cytology, and AI	92.9% accuracy, 95.7% sensitivity, 91.9% specificity, 0.966 AUC	AI was successfully able to differentiate malignant from benign pancreatic cystic lesions
Boas et al. (2022) [42]	Automatically diagnose mucinous cystic lesions using AI	98.5% accuracy, 98.3% sensitivity, 98.9% specificity, 1.00 AUC	Accurately differentiated mucinous from non-mucinous cysts

## Data Availability

The data presented in the study are openly available in online databases.
